# Can underwater endoscopic mucosal resection be an alternative to conventional endoscopic mucosal resection for superficial non‐ampullary duodenal epithelial tumors?

**DOI:** 10.1002/deo2.312

**Published:** 2023-11-03

**Authors:** Hidenori Tanaka, Yuji Urabe, Hiroki Takemoto, Kazuki Ishibashi, Hirona Konishi, Yuka Matsubara, Yudai Takehara, Shin Morimoto, Fumiaki Tanino, Noriko Yamamoto, Hajime Teshima, Junichi Mizuno, Issei Hirata, Hirosato Tamari, Akiyoshi Tsuboi, Ken Yamashita, Takahiro Kotachi, Hidehiko Takigawa, Ryo Yuge, Shiro Oka

**Affiliations:** ^1^ Department of Gastroenterology Hiroshima University Hospital Hiroshima Japan

**Keywords:** duodenal neoplasm, endoscopic mucosal resection, learning curve, superficial non‐ampullary duodenal epithelial tumor, underwater endoscopic mucosal resection

## Abstract

**Objectives:**

Underwater endoscopic mucosal resection (UEMR) is a simpler procedure for superficial non‐ampullary duodenal epithelial tumors (SNADET) than conventional endoscopic mucosal resection (cEMR). This study aimed to evaluate whether cEMR can be substituted by UEMR for SNADET in terms of effectiveness, safety, and learning curve.

**Methods:**

A total of 157 consecutive patients with 203 SNADETs ≤20 mm in diameter, including 107 lesions resected by cEMR and 96 lesions resected by UEMR, between January 2019 and May 2023, were retrospectively recruited. The treatment outcomes were compared between the cEMR and UEMR groups. The risk factors for incomplete resection by UEMR were analyzed using univariate and multivariate analyses. Lesions in the UEMR group were divided chronologically into five periods; thereafter, the en bloc resection rate and procedure time were compared.

**Results:**

No significant differences existed between the cEMR and UEMR groups in the mean procedure time (3.9 min vs. 3.6 min, *p* = 0.1380) or en bloc resection rate (91% vs. 94%, *p* = 0.4138). No perforation was observed in either group. Tumor size was an independent risk factor for incomplete resection using UEMR (*p* < 0.01). The history of biopsy was not associated with incomplete resection using UEMR. The en bloc resection rate of UEMR was 100% (20/20) in the first period and ranged from 90% to 100% over all periods.

**Conclusion:**

UEMR is safe and effective for SNADET ≤20 mm, regardless of a history of biopsy, and is easy to learn. Thus, UEMR can serve as an alternative to cEMR.

## INTRODUCTION

Although not definitively proven, duodenal adenoma can develop into adenocarcinoma via the adenoma–carcinoma sequence; therefore, resection is recommended for superficial non‐ampullary duodenal epithelial tumors (SNADET).^1^ Conventional endoscopic mucosal resection (cEMR), in which the lesion is resected using a snare after submucosal injection, has been reported to be a minimally invasive treatment effective for SNADET.^2^, ^3^, ^4^, ^5^, ^6^, ^7^

Recently, underwater endoscopic mucosal resection (UEMR), in which the lesion is resected using a snare with a lumen filled with water, was developed by Binmoeller et al.^8^ The lesion and surrounding normal mucosa are floated by filling water, making them easily grasped by the snare. UEMR is a simpler procedure than cEMR because it does not require submucosal injection; therefore, it is becoming increasingly popular. The favorable outcomes of UEMR for SNADET ≤20 mm have been reported, with an en bloc resection rate, postoperative bleeding rate, intraoperative perforation rate, and delayed perforation rate of 75%–100%, 0%–2%, 0%, and 0%–0.6%, respectively.^9^, ^10^, ^11^, ^12^, ^13^, ^14^, ^15^, ^16^ Thus, the effectiveness and safety of UEMR have been proven; however, its superiority to cEMR has not been proven. The results of the comparison between cEMR and UEMR are divided.^9^, ^10^, ^12^, ^15^, ^17^ In addition, the risk factors for incomplete resection by UEMR have not been well discussed. Although UEMR is simpler than cEMR, the acceptability and learning curve of UEMR for less‐experienced endoscopists are also unclear. UEMR, which is simpler than cEMR, can be an alternative to cEMR if it is as effective and easy to learn as cEMR.

This study aimed to evaluate whether cEMR can be substituted by UEMR for SNADET ≤20 mm by comparing the outcomes as well as analyzing the risk factors for incomplete resection and the learning curve of UEMR.

## METHODS

**FIGURE 1:**
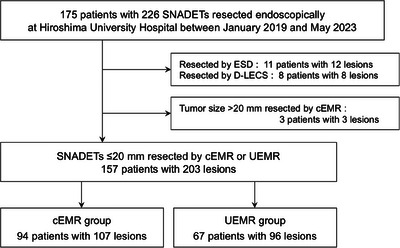
Flowchart of patient enrollment. cEMR, conventional endoscopic mucosal resection; D‐LECS, duodenal laparoscopic endoscopic cooperative surgery; ESD, endoscopic submucosal dissection; SNADET, superficial non‐ampullary duodenal epithelial tumor; UEMR, underwater endoscopic mucosal resection.

One hundred and seventy‐five consecutive patients with 226 SNADETs resected endoscopically at Hiroshima University Hospital between January 2019 and May 2023 were retrospectively recruited. Lesions resected by endoscopic submucosal dissection (ESD; *n* = 12) and laparoscopic endoscopic cooperative surgery (*n* = 8), as well as those >20 mm in diameter resected by cEMR (*n* = 3), were excluded. Therefore, a total of 107 lesions resected by cEMR (cEMR group) and 96 lesions resected by UEMR (UEMR group) were enrolled in the study (Figure 1).

Two expert endoscopists (HT and SO) with experience of >300 colonic EMRs performed cEMR between January 2019 and March 2021. UEMR was introduced to our hospital in April 2021, and since then, it has been performed by one endoscopist (HT) with no prior experience in UEMR. The cEMR and UEMR groups were divided according to the period before or after the introduction of UEMR.

The study was conducted in accordance with the Declaration of Helsinki, and the study protocol was approved by the Institutional Review Board of Hiroshima University (No. E2022‐0134).

### Procedure

For EMR and UEMR, midazolam was used for sedation, and butylscopolamine or glucagon was used to suppress peristalsis. A gastroscope with a water‐jet function (GIF‐Q260J or GIF‐H290T; Olympus Medical Systems) was used. For lesions located in the distal duodenum that could not be reached using gastroscopy, a colonoscope (CF‐H290TI; Olympus) was used. A transparent hood (TOP Corporation) was attached to the tip of the endoscope. Depending on the lesion size, a 10 or 15 mm electro‐surgical snare (Captivator II; Boston Scientific, or SnareMaster; Olympus) was chosen. For cEMR, 0.4% sodium hyaluronate (Muco Up; Boston Scientific) diluted twice with a 10% glycerin solution containing a small amount of indigo carmine was used for submucosal injection. For UEMR, the lumen was filled with saline using a water jet after air deflation. After the resection, the mucosal defect was completely closed using an EZ clip (Olympus).

### Evaluation

Treatment outcomes, including procedure time; en bloc resection rate; pathological complete resection rate, such as negative horizontal margin (HM0) resection rate, negative vertical margin (VM0) resection rate, and R0 (HM0 and VM0) resection rate; as well as the occurrence of adverse events, such as postoperative bleeding, intraoperative perforation, and delayed perforation, were compared between the cEMR and UEMR groups. Procedure time was defined as the time from the beginning of submucosal injection to the completion of resection for cEMR and from the beginning of water filling to the completion of resection for UEMR. The picture was taken just before the submucosal injection or water filling began and immediately after resection; therefore, the procedure time was calculated based on their time records. Non‐en‐bloc resection was defined as incomplete resection. Postoperative bleeding was defined as any apparent bleeding event. The histological diagnosis was categorized as adenoma, intramucosal carcinoma, or submucosal invasive carcinoma.

Risk factors for incomplete lesion resection in the UEMR group were analyzed using univariate and multivariate analyses. Lesions in the UEMR group were grouped chronologically into five periods to evaluate the learning curve of UEMR performed by a single endoscopist: cases 1–20, 21–40, 41–60, 61–80, and 81–96. The en bloc resection rate and procedure time were compared between periods.

### Statistical analysis

JMP version 16.0.0 (SAS Institute Inc.) was used for statistical analysis. Continuous variables were analyzed using Student's *t*‐test or the Mann–Whitney *U* test, and qualitative variables were analyzed using Pearson's chi‐squared test. A *p*‐value of <0.05 was considered statistically significant. Logistic regression analysis was conducted to examine the risk factors for incomplete resection by UEMR.

## RESULTS

The clinicopathological features of the enrolled patients and their lesions are presented in Table 1. Of the 96 lesions in the UEMR group, 2 (2%), 51 (53%), 40 (42%), and 3 (3%) were in the first portion, the oral side or concentric of the major papilla in the second portion, the anal side of the major papilla in the second portion, and the third portion, respectively. The mean tumor size was 8.1 mm in diameter. Seventeen lesions (18%) had a history of preoperative biopsy. The histological diagnosis after UEMR was adenoma in 84 lesions (88%) and mucosal carcinoma in 12 lesions (13%). No significant differences existed between the cEMR and UEMR groups in age, sex, tumor location, tumor size, history of preoperative biopsy, or histological diagnosis.

**TABLE 1:** Clinicopathological features of patients and lesions.

	Treatment method		
Variables	cEMR *n* = 107	UEMR *n* = 96	*p*‐value
Age, year, mean ± SD	57 ± 15	59 ± 13	0.5910
Sex, male (%)	65 (61)	65 (68)	0.3022
Tumor location (%)			0.3865
First	5 (5)	2 (2)	
Second oral	45 (42)	51 (53)	
Second anal	53 (50)	40 (42)	
Third	4 (4)	3 (3)	
On the fold (%)	87 (81)	76 (79)	0.7017
Tumor size, mm, mean ± SD	8.5 ± 3.7	8.1 ± 4.3	0.1291
Macroscopic type (%)			0.0054
0‐I	12 (11)	9 (9)	
0‐IIa	28 (26)	48 (50)	
0‐IIc	66 (62)	38 (40)	
0‐IIa + IIc	1 (1)	1 (1)	
History of biopsy (%)	31 (29)	17 (18)	0.0593
Histology (%)			0.4991
Adenoma	96 (90)	84 (88)	
Intramucosal carcinoma	10 (9)	12 (13)	
Submucosal invasive carcinoma	1 (1)	0 (0)	

Abbreviations: cEMR, conventional endoscopic mucosal resection; SD, standard deviation; UEMR, underwater endoscopic mucosal resection.

The treatment outcomes are presented in Table 2. No significant differences existed between the cEMR and UEMR groups in the mean procedure time (3.9 min vs. 3.6 min, *p* = 0.1380) or en bloc resection rate (91% vs. 94%, *p* = 0.4138). The HM0, VM0, and R0 resection rates were higher in the UEMR group than in the cEMR group, although the differences were not significant. Postoperative bleeding was observed in one case in the UEMR group, in which endoscopic hemostasis was achieved without blood transfusion. No perforation was observed in either group. Of the 16 cases with incomplete resection, the residual tumor was treated by additional ablation in 12 cases and additional snaring in 4 cases. Follow‐up endoscopy was performed in 7 cases, and local recurrence was not observed in all of them.

**TABLE 2:** Treatment outcomes.

	Treatment method		
Variables	cEMR *n* = 107	UEMR *n* = 96	*p*‐value
Procedure time, min, mean ± SD	3.9 ± 1.9	3.6 ± 2.2	0.1380
En bloc resection (%)	97 (91)	90 (94)	0.4138
Horizontal margin (%)			0.1940
Negative	60 (56)	65 (68)	
Unclear	42 (39)	29 (30)	
Positive	5 (5)	2 (2)	
Vertical margin (%)			0.9324
Negative	100 (93)	90 (94)	
Unclear	7 (7)	6 (6)	
R0 resection (%)	60 (56)	65 (68)	0.0889
Adverse event (%)			
Postoperative bleeding	0 (0)	1 (1)	0.2899
Intraoperative perforation	0 (0)	0 (0)	–
Delayed perforation	0 (0)	0 (0)	–

Abbreviations: cEMR, conventional endoscopic mucosal resection; SD, standard deviation; UEMR, underwater endoscopic mucosal resection.

**FIGURE 2:**
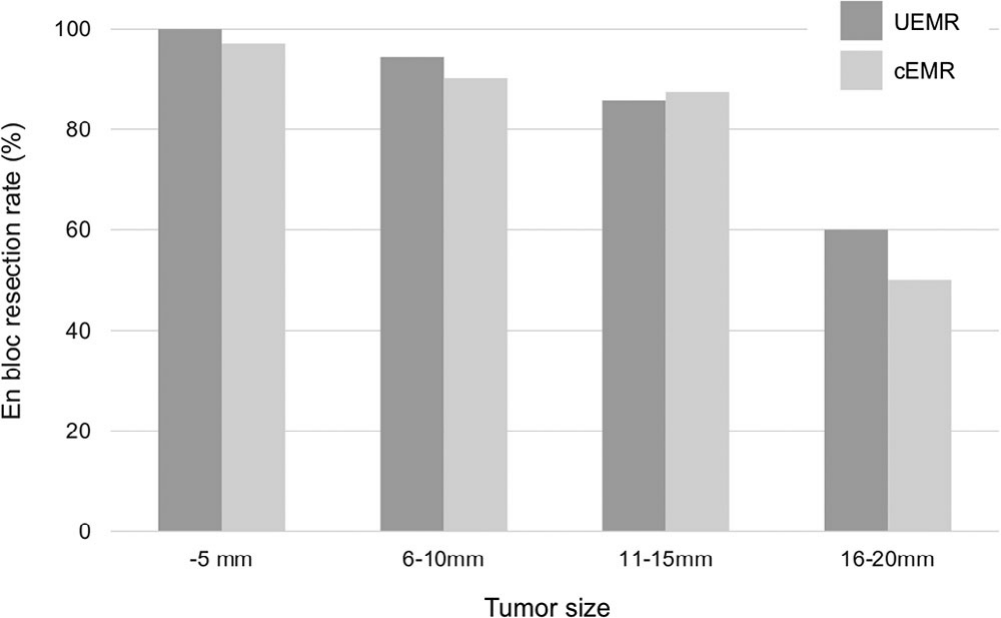
En bloc resection rate according to tumor size. The en bloc resection rates of underwater endoscopic mucosal resection (UEMR) and conventional endoscopic mucosal resection (cEMR) are represented by dark gray and light gray bars, respectively.

The risk factors for incomplete resection with UEMR were analyzed, as shown in Table 3. Tumor location on the oral side of the major papilla in the second portion (*p* = 0.04) and tumor size (*p* < 0.01) were risk factors for incomplete resection in univariate analysis. Multivariate analysis indicated that tumor size (*p* < 0.01) was an independent risk factor for incomplete resection. En bloc resection rates were 100% (41/41) for tumor size ≤5 mm, 94.4% (34/36) for 6–10 mm, 85.7% (12/14) for 11–15 mm, and 60% (3/5) for 16–20 mm (Figure 2). A history of biopsy was not associated with an incomplete resection.

**TABLE 3:** Risk factors of incomplete resection for superficial non‐ampullary duodenal epithelial tumors (SNADET) by underwater endoscopic mucosal resection.

Variables	Univariate analysis			Multivariate analysis		
	Odds ratio	95% CI	*p‐*value	Odds ratio	95% CI	*p‐*value
Tumor location						
First	Ref	–	–	Ref	–	–
Second oral	0.041	0.00–0.92	0.044	0.23	0.00–2.1 × 10^2^	0.67
Second anal	0.053	0.00–1.2	0.064	0.49	0.00–4.3 × 10^2^	0.84
Third	0.50	0.012–20	0.71	2.4	0.00–2.9 × 10^3^	0.81
On the fold	0.50	0.085–2.9	0.44	0.26	0.018–3.7	0.32
Tumor size (per mm)	1.4	1.1–1.7	0.0020	1.4	1.1–1.8	0.0020
Macroscopic type						
0‐I	Ref	–	–	Ref	–	–
0‐IIa	0.15	0.018–1.3	0.081	0.75	0.018–31	0.88
0‐IIc	0.19	0.023–1.6	0.13	1.8	0.045–73	0.75
0‐IIa + IIc	–	–	–	–	–	–
History of biopsy	2.5	0.42–15	0.31	1.5	0.10–23	0.76

**FIGURE 3:**
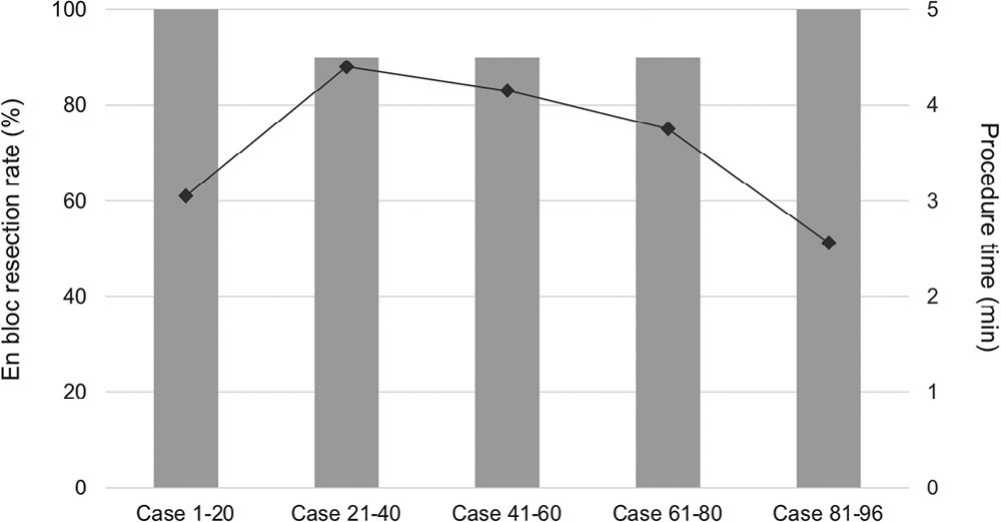
Chronological changes in en bloc resection rate and procedure time in the underwater endoscopic mucosal resection (UEMR) group. The bar chart shows the en bloc resection rate, and the line chart shows the procedure time.

Chronological changes in the en bloc resection rate and procedure time in the UEMR group are shown in Figure 3. The en bloc resection rate ranged from 90% to 100% over all periods. The mean procedure time during the initial phase was 3.05 min, followed by 4.40, 4.15, 3.75, and 2.56 min.

## DISCUSSION

Our study evaluated whether cEMR can be substituted by UEMR for SNADET ≤20 mm. Although randomization was not performed, the cEMR and UEMR groups were divided according to the period before or after the introduction of UEMR. As a result, UEMR for SNADET ≤20 mm is as safe and effective as cEMR. Tumor size was the only independent risk factor for incomplete resection using UEMR. The outcomes of UEMR were sufficiently good from the beginning of the introduction.

Although the outcomes of cEMR and UEMR for SNADET ≤20 mm in diameter have been compared in some published literature, those were all retrospective studies with a relatively small number of cases, and the superiority of UEMR has not been well established. Kuguchi et al. reported that the technical success rate of UEMR was significantly higher than that of cEMR (87% vs. 70%, *p* < 0.01). However, en bloc resection and R0 resection rates were significantly lower (87% vs. 95%, *p* < 0.01 and 67% vs. 80%, *p* = 0.05, respectively).^10^ The higher complete resection rate of cEMR could be due to the exclusion of difficult cases by converting to ESD. Furukawa et al. reported a higher en bloc resection rate and shorter procedure time of UMER compared to cEMR (96.4% vs. 72.2%, *p* < 0.05 and 4 min vs. 9.5 min, *p* < 0.05, respectively).^9^ A meta‐analysis also showed that UEMR could achieve a higher en bloc resection rate and shorter procedure time compared to cEMR.^17^ Perforation rarely occurred during or after the procedure.^9^, ^10^, ^11^, ^12^, ^13^, ^14^, ^15^, ^16^, ^17^ In this study, no significant differences existed in any treatment outcome between the cEMR and UEMR groups. The outcomes of cEMR were sufficiently good in this study, which could explain the lack of differences from those of UEMR. HM0/VM0 resection rates were higher, and unclear horizontal/vertical margin resection rates were lower in the UEMR group than in the cEMR group, although the differences were not significant. Underwater conditions might contribute to sufficient submucosal grasping by the snare and a reduction in thermal tissue damage by electric currents. Overall, en bloc and perforation rates of UEMR in this study were 94% and 0%, respectively, consistent with those in previous studies.^9^, ^10^, ^11^, ^12^, ^13^, ^14^, ^15^, ^16^

Tumor size ≥20 mm^11^, ^18^ and incomplete resection^6^, ^11^, ^19^, ^20^ have been reported as risk factors for residual lesions. Therefore, an adequate choice of treatment option to achieve en bloc resection is required for SNADET. Although ESD could achieve a high en bloc resection rate regardless of tumor size, it is technically challenging with a high risk of adverse events, such as perforation. Therefore, snaring methods, such as cEMR and UEMR, are safer for resecting SNADET ≤20 mm in diameter.^7^, ^21^, ^22^, ^23^

Few studies have evaluated the risk factors for incomplete resection using UEMR. Iwagami et al. indicated that among factors such as age, sex, tumor size, tumor location, and macroscopic type, only tumor size was associated with piecemeal resection.^11^ In addition to the same results as in previous studies, our study also indicated that a history of biopsy was not a risk factor for incomplete resection. Although biopsy should not be performed for SNADET that is endoscopically resectable because the accuracy of biopsy sampling has been reported as not superior to endoscopic findings due to the heterogeneity of tumors,^24^, ^25^, ^26^ it is still done in clinical practice. Although the lesions after biopsy are sometimes obliged to convert to ESD during cEMR due to submucosal fibrosis and non‐lifting signs induced by biopsy,^24^ our results suggest that UEMR can achieve en bloc resection for such cases, as in previous case reports.^27^, ^28^

Although UEMR is effective and simple, demonstrating its acceptability by less‐experienced endoscopists is important for its popularization. The learning curve of UEMR has not been well investigated; it could be evaluated because UEMR was performed by a single endoscopist in this study. No perforations were observed throughout the period. The en bloc resection rate was high, and procedure time was short from the beginning of the introduction of UEMR and was not affected by the accumulation of experience. These results strongly support the idea that UEMR is easy to learn and acceptable for less‐experienced endoscopists.

This study has some limitations. First, this was a single‐centered, retrospective study. Although the cEMR and UEMR groups were divided according to the period before or after the introduction of UEMR, which could reduce a selection bias, the potential for bias cannot be ruled out because this study was not a randomized trial. Second, the recurrence rate was not evaluated. Third, the learning curve demonstrated by a single endoscopist in this study may not be universal. Nevertheless, we demonstrated the substitutability of UEMR for cEMR for SNADET ≤20 mm.

In conclusion, UEMR is safe and effective for SNADET ≤20 mm, regardless of a history of biopsy, and is easy to learn. UEMR can be an alternative to cEMR for SNADET ≤20 mm.

## CONFLICT OF INTEREST STATEMENT

All authors declare no conflicts of interest for this article.
